# Severe iron-deficiency anemia as initial manifestation of pulmonary hemosiderosis in a child

**DOI:** 10.31744/einstein_journal/2018RC4505

**Published:** 2018-11-29

**Authors:** Natália Noronha, Pedro Ramalho, Rogério Barreira, Juliana Roda, Teresa Reis Silva, Miguel Félix

**Affiliations:** 1 Serviço de Cardiologia Pediátrica, Centro Hospitalar e Universitário de Coimbra, Coimbra, Portugal; 2 Serviço de Pediatria Médica, Centro Hospitalar e Universitário de Coimbra, Coimbra, Portugal; 3 Serviço de Pneumologia, Hospital Geral, Centro Hospitalar e Universitário de Coimbra, Coimbra, Portugal; 4 Serviço de Anatomia Patológica, Centro Hospitalar e Universitário de Coimbra, Coimbra, Portugal

**Keywords:** Anemia, Iron deficiency, Pulmonary diseases, Lung disease, Hemosiderosis, Child, Anemia, Deficiência de ferro, Pneumopatias, Hemossiderose, Criança

## Abstract

Idiopathic pulmonary hemosiderosis is a potentially fatal disease that results from episodes of alveolar hemorrhage of unknown origin. The clinical spectrum is varied, and anemia may constitute the only manifestation of illness, preceding other signs and symptoms by several months. We present the case of a 4 year-old child presenting with fever, vomiting and prostration, associated with pallor. He had microcytic and hypochromic anemia refractory to iron therapy. Gastrointestinal bleeding was ruled out after negative extensive etiological investigation. Subsequently, pulmonary infiltrates suggestive of alveolar hemorrhage were observed in the chest radiography. The cytological exam of the bronchoalveolar lavage showed hemosiderin-laden macrophages. After the etiological study, the diagnosis of idiopathic pulmonary hemosiderosis was made by exclusion. He was initiated on corticosteroid therapy, later associated to an immunosuppressive agent, with subsequent correction of anemia and of the radiological pattern. The patient is currently asymptomatic.

## RESUMO

A hemossiderose pulmonar idiopática é uma doença potencialmente fatal que cursa com episódios de hemorragia alveolar de etiologia desconhecida. As manifestações clínicas são variadas, e a anemia pode constituir o único sinal de doença, precedendo em vários meses os outros sinais e sintomas. Apresenta-se o caso de criança de 4 anos, com febre, vômitos e prostração, associados à palidez. Apresentava anemia microcítica e hipocrômica, refratária à terapêutica com ferro. A hipótese diagnóstica de sangramento gastrintestinal foi excluída, após investigação etiológica extensa, inconclusiva. Posteriormente, em radiografia torácica, foram observados infiltrados sugestivos de hemorragia alveolar. O exame citológico do lavado broncoalveolar mostrou macrófagos com depósitos de hemossiderina. Após estudo etiológico, assumiu-se, por exclusão, o diagnóstico de hemossiderose pulmonar idiopática. Foi iniciada terapêutica com corticoides, associada posteriormente a imunossupressor, com correção subsequente da anemia e do padrão radiológico, encontrando-se, atualmente, assintomático.

## INTRODUCTION

Pulmonary hemosiderosis (PH) is a rare disease that may occur at any age, although it generally affects children, with an estimated incidence of 0.24 to 1.23 case per million^(^ ^1^ ^,^ ^2^ ^)^ It is characterized by recurrent episodes of diffuse alveolar hemorrhage, manifested by hemoptysis. alveolar infiltrates on chest radiograph and variable degrees of iron-deficiency ferropenic anemia, resulting from loss of blood to the alveolar space. Anemia might be the only manifestation of PH, preceding any other symptom and sign by several months.

## CASE REPORT

A 4 year-old boy with a previous history of recurrent wheezing, with no criteria of severity and no associated manifestations, was observed in an emergency department for fever, vomiting and prostration. He had severe pallor, which prompted analytical investigation, that revealed microcytic and hypochromic anemia with hemoglobin (Hb) of 4g/dL, red cell distribution width (RDW) of 27% and 4% of reticulocytes, with no other relevant abnormalities. He was transfused with packed red blood cells and was admitted for treatment and investigation. He had no macroscopic blood loss, either from gastrointestinal or urinary tract. After 6 days, he was discharged on oral iron therapy, with Hb of 8.1g/dL and referred to Hematology for outpatient follow-up.

Five days after being discharged, he was seen again for fever, asthenia, coryza, and productive cough. He was pale, prostrated and tachypneic, with dispersed bilateral rhonchi on pulmonary auscultation. Analytically he had Hb of 2.8g/dL and marked anisopoikilocytosis on the peripheral blood smear. He was transfused with packed red blood cells and readmitted. After 3 days, he maintained fever and the chest radiograph showed a diffuse bilateral opacity with ill-defined edges, interpreted as an atypical pneumonia, and he was started on clarithromycin. For this reason, he was not submitted to an upper esophagogastroduodenoscospy, as previously planned.

**Figure 1:**
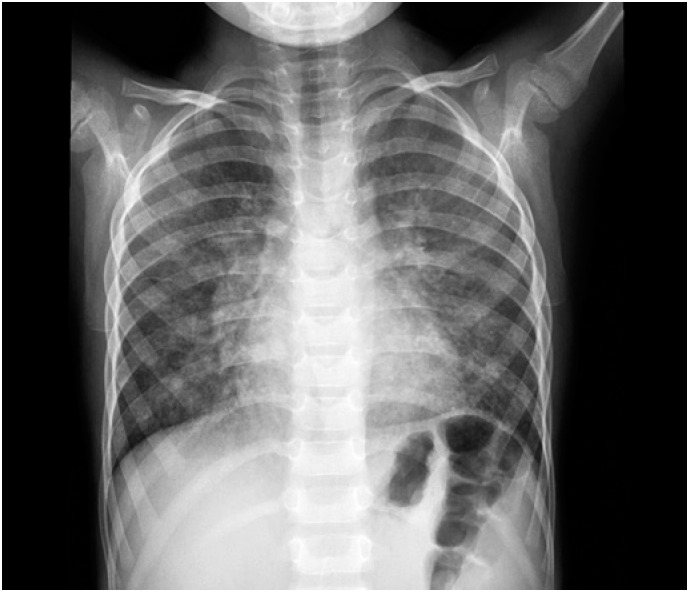
Chest X-ray. Diffuse bilateral opacity with ill-defined edges, with a predominantly central location

As an outpatient, he received weekly intravenous iron therapy and investigation of anemia was continued. Vitamin B_12_ and folic acid were within normal range and the bone marrow smear was compatible with sideropenia. Celiac disease, cystic fibrosis and pernicious anemia were excluded. The coproculture, parasite stool exam and fecal occult blood test were negative. As there was a poor response to intravenous iron therapy, he was readmitted to further investigate gastrointestinal bleeding. Upper and lower gastrointestinal endoscopies with biopsies were performed and no hemorrhagic lesion was found, and the mucosa was macroscopically and histologically normal. Abdominopelvic ultrasound, gastroduodenal and intestinal series, intestinal scintigraphy and enteral magnetic resonance imaging were normal. As such, given the iron-deficiency anemia with characteristics compatible with blood loss (although not identified), and despite the absence of respiratory symptoms at this phase, we started looking for other sources of hemorrhage, namely pulmonary. The chest radiograph showed a diffuse bilateral opacity with ill-defined edges, with a predominantly central location (Figure 1), suggestive of alveolar hemorrhage.

**Figure 2:**
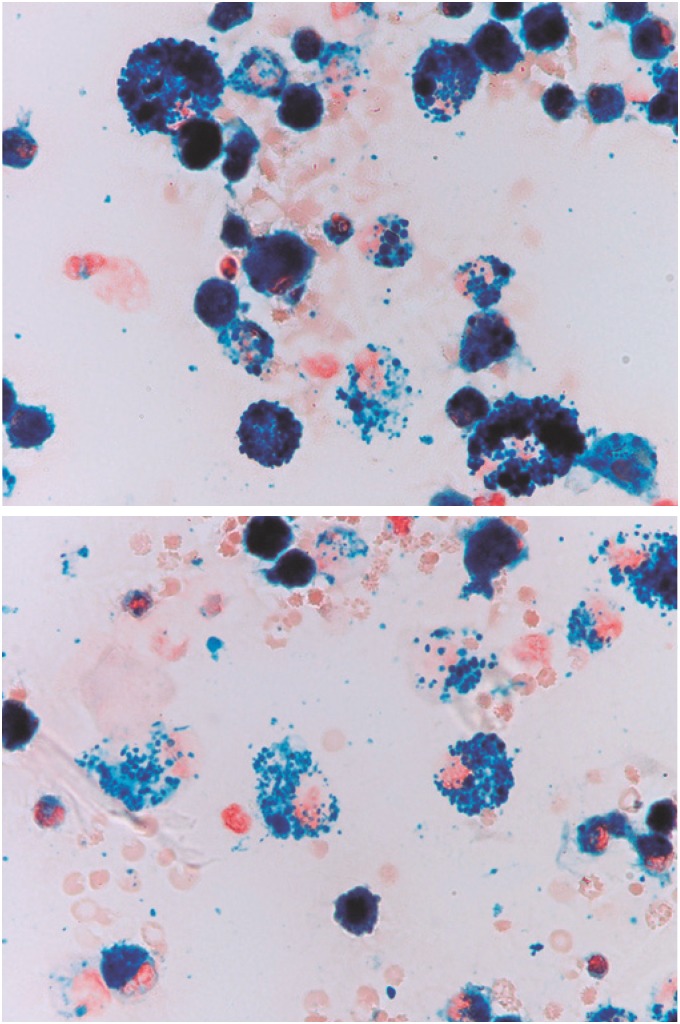
Bronchoalveolar lavage fluid showing numerous hemosiderin-laden macrophages stained positive with Perls

**Figure 3:**
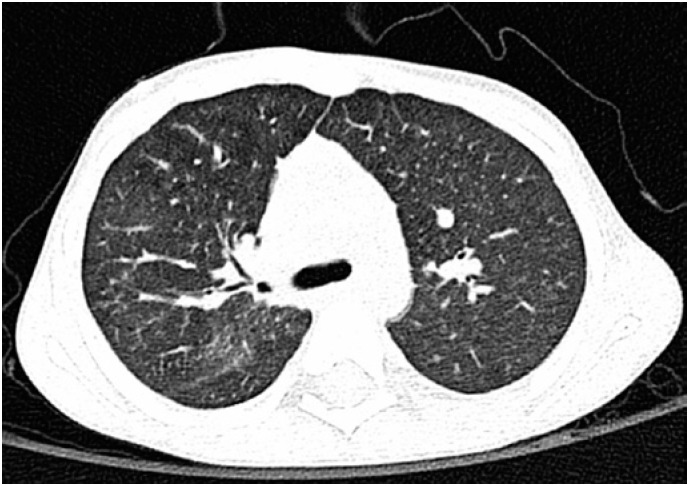
Chest computerized tomography scan. Diffuse increase of the parenchyma's density with no zone predominance, with dispersed areas of lower density, and several small dispersed nodules

To confirm alveolar hemorrhage, a fibrobronchoscopy was performed. There were no morphologic abnormalities or changes of the respiratory mucosa, and the cytological exam of the bronchoalveolar lavage showed 20.000/*μ*L erythrocytes. Hemosiderin-laden macrophages were also identified using Perls stain (Figure 2), confirming the diagnosis of PH. A pulmonary computerized tomography (CT) scan (Figure 3) showed diffuse ground-glass attenuation in both lungs with no zonal predilection, some dispersed areas of lower density, and several scattered micronodules. The patient was initiated on high dose methylprednisolone (20mg/kg/day, 3 days), followed by oral prednisolone. After excluding the most frequent causes of PH: auto-immune diseases (negative autoantibody profile), congenital cardiopathies, cow's milk protein allergy, among others - the diagnosis of idiopathic PH was made.

After starting steroid treatment there was a progressive increase in Hb level and improvement of radiological abnormalities. At follow-up, hydroxychloroquine was added (up to a dose of 5mg/kg/day) due to a drop in the Hb level coinciding with an attempt to taper steroid dosage. Subsequently, there was laboratory improvement and corticosteroid was tapered. The child is currently asymptomatic, with Hb levels between 11.3 and 13.3g/dL.

## DISCUSSION

Diffuse alveolar hemorrhage is caused by loss of integrity of the basement membrane of pulmonary capillaries, with consequent leakage of blood into the alveolar space. The low pressure and high capacitance of the pulmonary circulation result in chronic, diffuse and low-grade hemorrhage. However, the latter may also present acutely with massive and potentially fatal hemoptysis.

The gold standard for the diagnosis of diffuse alveolar hemorrhage is pulmonary biopsy, which may also enable the diagnosis of some of the specific causes of hemorrhage. Pulmonary hemorrhage can also be confirmed by the identification of hemosiderin-laden macrophages in the bronchoalveolar lavage fluid.^(^ ^1^ ^–^ ^3^ ^)^ This accumulation of hemosiderin confirms the role of macrophages in the removal of free erythrocytes from the alveolar space, with subsequent accumulation of iron in the form of hemosiderin. Although they do not make the definitive diagnosis of alveolar hemorrhage, chest radiography and CT scan may present suggestive (but not specific) aspects of hemorrhage, additionally contributing to the exclusion of other conditions.

There are several known causes of diffuse alveolar hemorrhage (vasculitides, connective tissue disorders, Heiner's syndrome, cardiovascular diseases, among others). However, diagnosis of idiopathic PH should only be considered when there is no other identifiable cause for the episodes of hemorrhage. Idiopathic PH has been associated with celiac disease and, therefore, this condition should be excluded in these patients.^(^ ^2^ ^)^

There are no universal recommendations regarding treatment of idiopathic PH.^(^ ^2^ ^)^ Corticosteroids are usually the treatment of choice in the acute phase of the disease.^(^ ^4^ ^–^ ^6^ ^)^ However, its role in the chronic phase remains unknown.^(^ ^2^ ^,^ ^4^ ^)^ Corticosteroids are associated with a reduction in the number of episodes of alveolar hemorrhage and may delay the progression to pulmonary fibrosis.^(^ ^1^ ^)^ Hydroxychloroquine and azathioprine are alternative immunosuppressants in refractory cases.^(^ ^1^ ^)^

Iron-deficiency anemia is the most frequent hematologic disease in pediatrics. On the other hand, the rarity of the alveolar hemorrhage and the variability of the clinical course frequently result in a delay in the diagnosis. In this situation, therapy is often started at the stage in which pulmonary fibrosis has already developed, with a higher risk of complications and worse prognosis.^(^ ^1^ ^)^ In our case, iron-deficiency anemia was the first and only sign of disease, leading to a delay in the diagnosis. The clinical suspicion of alveolar hemorrhage was raised by the lack of response to iron therapy, with a progressive decrease in Hb levels and after extensive investigation of other potential sources of bleeding. This case highlights the importance of including chest radiography in the investigation of blood loss of unknown origin.

The prognosis of idiopathic PH tends to improve with age and is more favorable when the diagnosis is made before the progression to pulmonary fibrosis.^(^ ^1^ ^)^ Chest CT scan has a prominent role in the early identification of fibrosis, and in our case, it contributed to the exclusion of this complication. In the past two decades, a 5-year mortality reduction from approximately 50% to 14%^(^ ^4^ ^)^ was observed, possibly due to the use of more intensive therapeutic regimens.^(^ ^3^ ^)^ Episodes associated with major bleeding usually have a worse prognosis,^(^ ^4^ ^)^ both in the acute and chronic phases of the disease.

## CONCLUSION

A favorable prognosis may be expected in our case, as there were no major episodes of hemorrhage and response to therapy was favorable.
